# Extra Supplement—The Nursing Mirror

**Published:** 1889-08-17

**Authors:** 


*The Hospital,*
AUGUST 17, 1889.

### The Nursing Mirror.

*Extra Supplement*

All Contributions for this Supplement should be addressed to the Editor, THE HOSPITAL, 139-140, Salisbury Court, Fleet Street, London, E.C., and should have the word "Nursing" plainly written in left-hand top corner of the envelope

### En Passant.

DOLL COMPETITION.—Our thanks are due to the many nurses who have sent in their names for the doll show. Some of them raise difficulties. Thus, one nurse wants to know how miniature scissors and forceps are to be got for chatelains. Several toy shops have miniature scissors, and forceps could surely be cut out of silver paper and glued together. It is the set of three dolls which will take the prize, not one separate doll. We will gladly answer any queries, or publish any suggestions, as we think a show of dolls in the uniforms of all the different hospitals will be a most interesting and very pretty sight.

VANCOUVER.—From what far-off lands do we get news through our kind correspondents! Here is a letter arrived from Vancouver Island with an account of St. Luke's Home there. Sister Francis, the matron, is very much liked, and is very energetic. She earns about sixty dollars a month by attending outside cases in midwifery, and all this goes to the home. There is a nice little church attached to the home, and two priests hold services there, besides doing all sorts of good work in the town. The home is liberally supported, but many nursing comforts and useful articles are wanting in the wards. The servants are Chinese—all servants are in Vancouver. The scenery is exquisite, and the ferns and flowers luxurious and beautiful; but, alas! the domestic fly is a terrible nuisance, and even at Vancouver roses have thorns!

SISTERS' UNIFORM.—Amongst the changes inaugurated by Miss Gordon, at Charing Cross, is cotton dresses for sisters, as well as nurses and probationers. The absurdity of permitting the head nurse to wear dresses which will not wash, while the other nurses are clothed in washing materials, is very evident. "Handclasp" wrote us a clever letter on this subject, which we printed in May. We commend the courage of Miss Gordon in thus starting such a sensible reform. The sisters' dress at Charing Cross is, of course, distinctive from that of the nurses or probationers. Both nurses and sisters have strings to their caps, but the probationers have none; their uniform is a red and white stripe, and very small neat cap. The outdoor uniform at Charing Cross now resembles Liverpool, save that the red hood is left out. All things are working smoothly with the new nursing staff.

SALVATION ARMY NURSES.—This is what Mrs. Bramwell Booth says about nurses: "We propose if possible to work a nursing institution in connection with the hospital to train and send out as nurses thorough practical, godly women, who will all be total abstainers, and, we hope, free from the 'professional' spirit, which seems to make many nurses now available anything but agreeable visitors to ordinary households." Mrs. Booth has here hit on one of the chief faults of private nurses, that they sometimes forget that they are women first, and so permit the professional spirit to kill the womanly spirit. It is exceedingly hard for a nurse to remember that every-day facts to her are startling and unpleasant to her patients; it is hard to remember that every patient is a human being firstly, and a case secondly. If Mrs. Booth has the sense to see the evil of the professional spirit in ordinary nurses, we hope she will also see the evil of training her nurses to minister to the soul first and then to the body. This is the fault which will mar Mrs. Booth's excellent scheme unless she is very careful.

ERGOT.—Some time ago a correspondent asked us a question about ergot which was never answered, but the proper reply to which seems to be the sentence of Dr. Pinzoni: "Ergot is a prophylactic against puerperal fever, and an indirect antiseptic agent."

VISITORS TO CAMBRIDGE.—For the amusement of our readers we give the following list of visitors to Cambridge on July 30: Mrs. Cole, Mrs. Fenwick, Mrs. Humphry, Mrs. O'Niell, Miss Baldwin, Miss Barton, Miss Cooper, Miss Harvey, Miss Homersham, Miss Kavanagh, Miss Kenealy, Miss Lohr, Miss Meyrick, Miss Sutcliffe, Miss C. J. Wood, Miss Young, Dr. Gage Browne, Dr. Bedford Fenwick, Dr. Humphry, Dr. Kenealy, Dr. Macalister, Dr. Bezley Thorne, Miss Furley, Mr. Pickering Pick, and about fifty more.

WORK IN INDIA.—Mrs. Sheppard, late of Poona, is the subject of an interview in the *Women's Penny Paper*, and various interesting details of her life are given. It was in Poona, where so much of her time was spent that Mrs. Sheppard initiated and carried through a reform, with which her name will always be associated, and the benefits of which are incalculable. Eight years ago there was not a single nurse in the Poona Hospital; there were only two ward boys and a few dirty and unskilful ayahs to attend upon some 150 patients. After the most persistent efforts Mrs. Sheppard succeeded in getting two European nurses appointed. After another three years, she was enabled to arrange for the reception of two sisters from Wantage. She asked Government to provide quarters for them, and after considerable delay, these were built and opened by Mrs. Sheppard, the furniture having been provided by an appeal to public generosity. In 1887, the staff was supplemented by four trained nurses from London hospitals, and at last, after untiring effort and considerable expenditure, the hospital has a staff of three sisters, four nurses, and one probationer—eight in all. Mrs. Sheppard is a life member of the Lady Dufferin Fund, and it was owing to her efforts that branches of the National Indian Association were started in the Bombay district in 1886. Mrs. Sheppard now resides near Regent's Park.

NURSING AT ASHTON.—Sir William Brooks lately took the chair at a meeting held at Ashton-on-Mersey to forward a scheme for district nursing. The circular setting forth the scheme states that it is intended to provide a trained lady nurse, whose time will mainly be spent in visiting the sick in Ashton, but any spare time will be devoted in the discretion of the managing committee to cases in the adjoining parishes of Sale, Brooklands, and Carrington. Secondly, to take a cottage for the nurse to live in and prepare food for the sick. One room will be reserved for patients. The following ladies comprise the committee for the first year: Mrs. Chetwynd Atkinson (secretary), Mrs. T. Craven, Mrs. Greenleaves, Mrs. B. Jackson, Mrs. C. Renshaw, Miss Scott, and Mrs. Windsor. The amount required per annum is estimated at £150. At the meeting, Dr. Charles Renshaw said he felt it a privilege to speak on behalf of the poor of Ashton. There was a vast difference between skilful nursing and that of ordinary individuals, and he illustrated this argument by narrating incidents in which want of means, or lack of knowledge, had intensified disease or caused death. He had great pleasure in moving the following resolution: "That the scheme of nursing the sick in this parish meets with the cordial approval of this meeting, and this meeting pledges itself to do all in its power to carry out the scheme." Dr. Withers and Dr. Israel Renshaw also supported the resolution.

### Physiology for Nurses.

### NO. VI.—ON THE SKELETON.

The skeleton is the framework to which the muscles and flesh are attached, and besides this it enables you to move and protect many delicate and very important organs, such as the heart, and lungs, and the brain. The skeleton is made up of more than 200 separate bones, which are for the most part as perfect in a child as in an adult, only of course on a smaller scale in the child. The skeleton is commonly divided into the head, trunk, and upper and lower limbs—the head consists of the skull and the face, that is 22 bones in all; the trunk is the central part, and is made up of the collar-bones, shoulder-blades, ribs, spine, and hip bones; the upper limbs are the arms, the upper part above the elbow, and the lower part below, the small bones at the wrists and the fingers with their joints. The lower extremities are the upper and lower parts of the leg, the knee, the bones at the ankle and the toes. To begin with the head. Unless you examine the skull very carefully, you would think it was all one bone, rounded at the top, and flat at the sides; but this is not really so; and if you look very carefully at a skull which has no skin on it, you will see wavy indented lines, like the teeth of two saws, so cut that the teeth of the saw on one side fit into those of the other most accurately, and thus, to all intents and purposes, there really is no division, and the bones of the skull in the adult are practically one. In the skulls of very old people these marks or "sutures," as they are called, disappear altogether. In the baby these various bones are not joined into one hard mass, otherwise the brain could not grow, and the baby, if it was to have the amount of brain due to it when older, would have to have a very large head in proportion to the rest of its body. As some of you, perhaps, know, in at least two places in the infant's skull there are soft places without bone, merely protected with the strong membrane which covers the whole of the brain, and of course the outside skin. If you look carefully you can see those places throbbing; that is the brain below; it throbs equally fast in all our skulls, only as the bones in our cases are united we cannot see it, but can feel it distinctly. Of course, with the brain only partially protected, rough handling or a blow might do great injury, and even kill the baby. Sometimes you hear of nurses who think themselves cleverer than Nature, and who press the bones of a baby's head together because they think it is a mistake of Nature's to have them apart. Perhaps some of you may have friends who have what is popularly called "pressure of the brain," and know the inconvenience of it, so you can realise to some extent how much the baby must suffer from this mistaken kindness of the nurses.

Another point which those who have the care of very young children must remember is that a baby must be sometimes nursed in one arm, sometimes in the other, or else its head may be deformed by the whole weight of the brain pressing on one side of the soft skull. In an adult the skull is very hard, and is so formed as to possess the greatest possible strength; that is the reason why the skull is more or less round, because that shape has been found to possess most resistance. This fact is also shown by the engineers' choice of a round form for bridges, tunnels, etc. The strength of the human skull is shown by the weight that people can carry on their heads without fear of breaking them in. Now you will ask why the skull should be made so strong, for you will naturally say it is not necessary for people to carry weights on their heads; there are other ways of carrying them besides that. The reason why the skull is so very strong is on account of the very important organ found inside—namely the brain—on which our power of movement depends, besides all sense, such as smell, sight, taste, etc., so that if the whole of the brain were destroyed entirely we should die. Though grown-up people can bear heavy weights on their heads children cannot. The shape of a baby's head can very easily be altered, and this fact is well known to many barbarous people.

For instance, some Indian tribes, living in the North of America, are not contented with the shape that Nature gives their babies' skulls, but think they can alter them and make them more beautiful, and, therefore, they do not mind treating their babies very cruelly. They put the poor little thing into a long narrow tub, and over its head they place a piece of hard stuff, to which strings are fastened, and then, for several weeks, these strings are fastened down firmly to the tub, and gradually the forehead goes nearly flat, and the head is much larger from back to front. Another tribe of Indians fasten a heavy tile on to the infant's head, which has exactly the same effect, and flattens it on the top. I am sorry to say that these poor ignorant people are not the only persons who give infants unnecessary pain by putting weights on their heads. The Indians know no better, and think they are doing a kindness by increasing the child's beauty according to their own ideas, but people of the present day ought to know better than to put such heavy bonnets and hats on their babies' heads, and the milliners ought not to be so ignorant as to make them, though if no one would buy them they would soon not be made any more. When next any of you have to buy a baby's bonnet, I hope now the first thing you think of will be the weight. Remember that what seems at all heavy to you as you hold it in your hand will be far too heavy for the baby to bear on its little soft skull. Even if this weight is not enough to alter the shape of the head, still it is quite enough to tire the head, not to speak of permanent harm to the brain in a way of which the parents have no idea at the time, though in after-life it may make him less clever and useful, and if he has to support a wife and children by his brain, this might be a very serious matter. No child must have any pressure on its head before it is two years old. You will now have some notion how important the skull and its contents, the brain with its control over the special senses of seeing, hearing, etc., is, and will be able to grasp how dangerous a blow on the head may be. Unfortunately, both men and women get very angry with each other, and lose all control over themselves, and their one wish being to injure or hurt one another, their knowledge of the danger there is in a blow on the head may make them choose that very part. A heavy blow on the top of the head might make a person lose their sight and memory for life. A severe box on the ear may make you deaf for the rest of your life. A young man of my acquaintance, many years ago, when a mere boy, tried to defend a poor woman who was being struck by her husband, and he received the blow which was intended for the unfortunate woman on his ear, and never entirely regained his hearing.

*(To be continued.)*

### CHEERFULNESS.

"To your sympathy and strength you must add cheerfulness. In a large degree the prevailing feeling in the sick-room will be bright and cheerful, or gloomy and dark, according as you make it. And here you will often find sore need for all your tact and ability. How with a tender sympathy for the suffering, and with a knowledge, perhaps, that the rest of those around you may not have, that greater sufferings and sorrow must follow in the course of the disease, how to infuse into the sick-room that intangible, yet potent influence, that shall pervade all that is said and done, so that there shall be brightness and hope, that is a task that is, indeed, often arduous; yet it is in the power of each one of you to do just these things."—*Dr. A. R. Paine.*

### The Nurses' Home, Sydney.

As so many of our friends have lately gone to Sydney we propose to give some notion of the life awaiting them there. Sydney is the most English of Australian towns in manners and habits, but it lacks the "go" of Melbourne. It possesses a lovely harbour and unrivalled botanical gardens, but it is 500 miles nearer the equator than Melbourne, and so is hotter and more cursed with flies and mosquitoes. The Nurses' Home, to which Lady Samuel and Miss Campbell have lately been despatching nurses from home, is at 140, Phillip-street, and is a comfortable building superintended by Miss Pinkerton, who trained at Crumsal Hospital, Manchester. She has thirty nurses under her, amongst whom we may mention Nurse French, once of the London Temperance; Nurse Herbert and Nurse Coote, once of the Middlesex; Nurse McKay, Nurse Bennet, and Nurse Tilley, once of Glasgow Royal Infirmary; Nurse Beasly and Nurse Thornbury, of Birmingham; and many others.

The fifth annual meeting of the home was held on the 14th of December, Sir Alfred Stephen, G.C.M.G., presiding, when the report for the preceding year was read, and the committee were re-elected. Lady Carrington was good enough to attend, and at the request of the committee presented the bonuses which had been awarded to Nurse Munro and Nurse French, for long and faithful service to the institution. A similar bonus was afterwards awarded to Nurse W. O'Toole. Another member of the staff received an appointment during the year: Nurse Munro becoming matron of the Goulburn Hospital, after five years of most satisfactory work at the home. She was trained at the Royal Free Hospital, London, and has had a great amount of experience in the profession.

A limited number of thoroughly-trained nurses are received into the home as resident nurses. The salary is at the rate of £26 per annum, and an additional 10s. per week while on active duty, or £1 per week in infectious cases, making a total of from £40 to £50 per annum. Board, lodging, washing, and uniform are provided. A few young women are annually trained as professional nurses, who become probationers in one of the hospitals of the colony, where they remain for either one or two years, and receive gratuitous board, lodging, and training, with an allowance towards clothing. At the end of the period of training, they serve the home as resident nurses for either one or two years, as may be agreed upon. The charge for the nurses' services is the same as in England, £2 2s. for ordinary cases and £3 3s. for infectious cases.

The rules are fairly strict. This is the first: "While not on active duty, each nurse will live at the home. She is to yield cheerful obedience to the matron, and to perform such domestic duties as may be assigned to her. She is to wear the prescribed uniform, to attend regularly at meals, and lectures or lessons, and must not leave the house without permission, but she will be expected to take regular outdoor exercise, and to attend the sick poor when called upon."

Sometimes nurses who join the home are disappointed at colonial ways. No one should emigrate who is not prepared to meet difficulties, which may be trivial, but are very irritating. As a rule colonials are very jealous of Englishwomen, yet they like and admire them and will work with them on terms of equality, but not under them. They are sensitive to a degree, and resent being patronised. The expensiveness of service in Australia makes it necessary for ladies to do more menial work than at home. This makes them more surprised when nurses object to sweep or dust. Very often nurses seem to think they will get less work and more pay in the colonies; but we doubt the less work, except in India, where lazy nurses can leave much to natives if they choose. In Sydney, at least, the work is hard.

### Everybody's Opinion.

*[Correspondence on all subjects is invited, but we cannot in any way be responsible for the opinions expressed by our correspondents. No communications can be entertained if the name and address of the correspondent is not given, or unless one side of the paper only be written on.]*

### THE PENSION FUND.

Nurse F., Bideford, writes: I think the suggestion of Nurse Barbara which appeared in your issue of the 27th ult., is an admirable one, and I shall have much pleasure in sending a trifle to the Bonus Fund when sending my premiums. I quite agree with her that the gratitude of all nurses is due to those gentlemen who have so generously interested themselves in making the Pension Fund a success, and have given such practical proofs of their sympathy.

Elizabeth writes: After reading the account in this week's Hospital of the National Pension Fund for Nurses, I hasten to offer my sincere and most grateful thanks to you and all who have so kindly interested yourselves on behalf of us nurses. I would like to give my thanks more especially to those four gentlemen, Lord Rothschild, Henry Hucks Gibbs, Esq., E. A. Hambro, Esq., and Junius S. Morgan, Esq., who gave the £20,000 to start the Pension Fund, which, I think, must prove the greatest boon that could have been conferred upon any profession. And I am sure their further generosity in giving another £5,000 for the special benefit of the first 1,000 nurses (of whom I am one) will win our deepest gratitude. It is indeed most gratifying to know that we have so many kind friends interesting themselves in our behalf. There is no doubt that nursing is a noble profession, and I sincerely hope we shall always prove ourselves worthy of the kindness and generosity conferred upon us by the promoters of the National Pension Fund.

Nurse Emily Hayes writes: Allow me through the Hospital to express my thanks to you and the many City merchants who have so kindly brought the Nurses' Pension Fund to what it is, amidst so much opposition and so many difficulties. I feel not any of us can express all we would for your very great kindness and the amount of trouble you have taken; and the generous gifts of the gentlemen in helping us to provide for sickness and old age. Please accept my very best thanks for your disinterested kindness, and the gentlemen for their noble generosity.

S. E. A. writes: I should like to take this opportunity of thanking those gentlemen whose kind thoughts and magnificent generosity has been bestowed so liberally upon the nurses of England. I cannot find words that will adequately express my feelings of gratitude on behalf of myself and sister nurses; but I am sure many will be the sincere thanks that will be given to those who have so substantially helped nurses to provide for themselves in sickness and old age. With sincere thanks from one solitary nurse who appreciates the movement.

[Many similar letters have been received, which we have no room to publish.—Editor.]

### THE SUNDERLAND SISTERS.

District Nurse writes: I think you would like to know that the subject of the payment of the sisters of Sunderland Infirmary was discussed at the late annual meeting. Dr. Morgan said a resolution had been placed in his hand which he considered to be of great importance. For some years the committee had had under their consideration the fact that the sisters who were doing such noble work there had no provision made for their old age and infirmities. Many misunderstandings had arisen as to their position with regard to those sisters, and he thought it would be right that the public should be placed in possession of the facts. Some sixteen or seventeen years ago a committee from the institution visited London in order to find out the most suitable system of nursing. They went to various hospitals, and, after due enquiry, came to the conclusion that the nursing sisters sent out by the Tottenham Institute were the most likely to fulfil all their requirements, and acting on the recommendation of that sub-committee the committee of the infirmary made arrangements with Dr. Lazeron for seven sisters to come down, and these had since been the nurses at the infirmary. It was rather a difficult thing to explain exactly their financial position. The infirmary had paid to the institution which trained them an annual sum. That was at first £250. Not one shilling of that went to the sisters. It went to the institution which trained them. Eventually the sum was raised to £400, at which it now stood, but the sisters individually got nothing from the infirmary and their services were rendered gratuitously. Their pay from Tottenham was £8 a year each, and that was all they got. Dr. Morgan went on to propose that a Provident Fund should be started for the sisters, and the strange point is he seemed utterly unaware of the existence of the Pension Fund, and what much better terms it could afford to give to the devoted sisters who are loved and admired by us all.

ASYLUM ATTENDANTS.

A. B. C. writes: Once more we attendants have been made to feel that even a tragedy committed in our first-class district asylum—namely, the Richmond, Dublin City—only called for a strong protest against the managers by some jurors. As an experienced asylum attendant, I must acknowledge the system of nursing the insane calls for prompt and thorough investigation. What training do we receive in England or Ireland? Learn all you can for yourself. No doctor ever gives nurses a lecture, no head attendant ever instructs, no books are given for study. And yet we are supposed to act as medical nurses, though often some of us do not know how to put on a poultice. We do our best, as we should do in our homes, but it ends there. A lady, hospital-trained, should go with the doctors to lectures, and train all attendants. Mental nursing should hold a first place in the profession.

### Appointments.

[It is requested that successful candidates will send a copy of their applications and testimonials, with date of election, to THE EDITOR, The Lodge, Porchester-square, W.]

CHICHESTER INFIRMARY.—The inhabitants of Chichester in general, and the infirmary and Dr. Bostock in particular, are to be congratulated on the selection of Miss Wyld for the post of matron and lady superintendent of the nursing branch. Under her management we believe it will be impossible that any nurse will be sent out from this institution to take charge of a sick infant, who will be found administering narcotics from a bottle brought with her. Miss Wyld, trained in the Nightingale School, became head nurse at the St. Marylebone Infirmary in 1884, was promoted to be second assistant matron there six months later and placed in charge of the nurses and probationers four months after. In 1887 she became assistant matron of the St. Marylebone Infirmary, and undertook the duty of preparing the probationers for the medical superintendent's lectures and for the annual examination. Miss Wyld has received several gratuities for faithful discharge of duties from the Nightingale Fund, including a special one as a mark of the Managers satisfaction with the manner in which she instructed the probationers. For the last eighteen months she has been matron of the Pavilions, Darenth Asylum. Five and a half years ago Mrs. Wardroper declared that good common-sense pervaded all Miss Wyld's work, and her connection with the Chichester Infirmary will doubtless prove as grateful to the managers and all concerned as it will be gratifying to herself.

### TO PRIVATE NURSES.

"FROM this time all your patients will be your employers, and they and their friends will expect and demand a certain amount of respect and subserviency; see, then, that you render them. Beware of wearying of the cares that belong to those *chronically* ill, or to those who may be passing through the tedium of a slow convalescence. As long as there is danger to life, I know you will be ever constant in your care, but the monotony of convalescence will try many of you sorely. Your charge may then become exacting, and with it fretful, and remember, this is something to which you have been unaccustomed, for your patients heretofore have belonged to that class too poor and humble to indulge successfully in such luxuries."—*Dr. W. M. Polk.*

### Winter Amusements and Remunerative Work.

AN EXHIBITION OF DOLL NURSES.

THE EDITOR of THE HOSPITAL has much pleasure in offering to nurses prizes to the value of £10 for the three best dressed sets of dolls representing the nursing staff of any hospital. The dolls must not be shorter than four inches nor taller than twelve inches, and must represent the sister, charge-nurse, and probationer of the hospital. Any nurse intending to compete must send her name and address on a post-card, headed "Doll Competition," together with the name of the institution or hospital, the uniform of which is to be faithfully reproduced, to the Editor of THE HOSPITAL, The Lodge, Porchester-square, London, W. *before September 1.* Further particulars will be announced later on, and arrangements will be made for a public exhibition of the dolls where nurses will be able to see the show. The number and amount of the prizes will not be fixed at present in deference to a general wish, and suggestions on this point are invited.

### Vacancies.

The following vacancies are announced:

**Matrons.**

Swansea Hospital, £50.
Victoria Infirmary, Glasgow, £70

**Nurses.**

Beckett Hospital, Barnsley, £18.
Birmingham Orthopœdic Hospital.
Dulwich Infirmary (several), £20.
Guernsey Cottage Hospital, £25.
Halifax Fever Hospital, £18.
Hanwell Ophthalmic School (one charge, £26; six assistant, £18).
Hospital for Sick Children, Stepney (three), £25.
Kent Nursing Institution.
Kingston Home, Hull.
Liverpool Stanley Hospital, £25.
Miss Armstrong's Home, Ventnor.
Miss Stewart's Institution, Glasgow.
National Hospital, Queen Square, £22.
Newcastle Nurses' Home (two), £25.
North-Western Fever Hospital, £30.
Nurses' Institute, Jersey, £25.
Pendlebury Hospital, £20.
Queen Victoria Institute, Wolverhampton.
Rochdale Infirmary, £22.
Rotherham Hospital (assistant), £16.
Seamen's Hospital, £20.
Torquay Sanatorium, £30.
Victoria Home, Bournemouth.
Weston-super-Mare Nurses' Institute.
Workhouse Infirmary Nursing Association (several).

**District Nurses.**

Pembroke.
Penzance, £40.
Queen Victoria Institute, Edinburgh.

**Probationers.**

Bolton Infirmary.
Brighton Fever Hospital.
Burnley Hospital (several).
Carmarthen Infirmary, £8.
Great Yarmouth Hospital.
Workhouse Infirmary Nursing Association (several).

### Notes and Queries.

NOTICE.—We must really ask our correspondents to be more particular in enclosing name and address. Constantly we receive queries or letters with no name attached.

**Queries.**

(66) *Home Wanted.*—A comfortable home wanted for a bedridden old lady who cannot pay more than £30 a year. *C. M. H.*

(67) *Private Nursing in Canada.*—Can any of your readers give me the address of a private nursing home in the States or Canada? *H.*

(68) *Brisbane.*—Is there any English agent for nursing in Rockhampton or Brisbane? *No. 12.*

(69) *India.*—A nurse who is going to Bombay would be very glad of particulars of outfit which will be necessary. Is the usual uniform worn by nurses in India? *Bombay.*

**Answers.**

*Probationer.*—Should apply to the Matron, Central London Ophthalmic Hospital, 238, Gray's-inn-road, London, W.C.

*S. B. H.*—See answer to query (65) last week.

*Stretcher.*—After satisfactorily passing three of the St. John's Ambulance examinations, a badge (a small cross) is given which can be worn on the watchchain, but not on the breast as a medal.

*M. Cross.*—A year's workhouse infirmary training will not admit you into the Army or Navy staff of nurses, and few hospitals will regard you as a trained nurse under those circumstances. You require a certificate before you can be regarded as a trained nurse.

*Agnes Page.*—It is impossible for us to advise probationers to apply at one hospital rather than at another. Any hospital near you which has 100 beds is sure to offer you good training. A probationer usually binds herself to serve the hospital for three years. She gets £10 the first year, and perhaps £15 or £20 the other years. Any matron will give you full particulars if you apply to her.

